# Responses in the left and right entopallium are differently affected by light stimulation in embryo

**DOI:** 10.1016/j.isci.2024.109268

**Published:** 2024-02-19

**Authors:** Giacomo Costalunga, Dmitry Kobylkov, Orsola Rosa-Salva, Anastasia Morandi-Raikova, Giorgio Vallortigara, Uwe Mayer

**Affiliations:** 1 Center for Mind/Brain Science, University of Trento, Piazza Manifattura 1, Rovereto, 38068 TN, Italy

**Keywords:** Physiology, Sensory neuroscience

## Abstract

Sensory stimulation during the prenatal period has been argued to be a main factor in establishing asymmetry in the vertebrate brain. However, though largely studied in behavior and neuroanatomy, nothing is known on the effects of light stimulation in embryo on the activities of single neurons. We performed single-unit recordings from the left and right entopallium of dark- and light-incubated chicks, following ipsi-, contra-, and bilateral visual stimulation. Light incubation increased the general responsiveness of visual neurons in both the left and the right entopallium. Entopallial responses were clearly lateralized in dark-incubated chicks, which showed a general right-hemispheric dominance. This could be suppressed or inverted after light incubation, revealing the presence of both spontaneous and light-dependent asymmetries. These results suggest that asymmetry in single-neuron activity is present at the onset and can be modulated by environmental stimuli such as light exposure in embryos.

## Highlights

• Light incubation increased the responsiveness of visual neurons in the entopallium
• Entopallial responses are lateralized in dark-incubated chicks
• Asymmetry in single-neuron activity is present at the onset of life
• Spontaneous lateralization can be modulated by environmental stimuli in the embryo

## Graphical abstract

**Figure:**
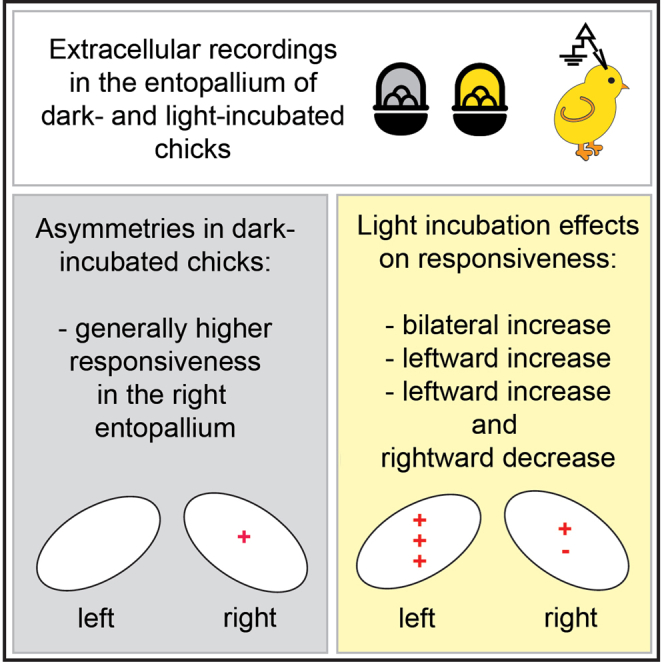

## Introduction

Sensory stimulation during embryogenesis affects cognitive development in areas ranging from human language development^1^ to inferential reasoning and social coordination in animals.^2^^,^^3^^,^^4^^,^^5^^,^^6^ Research with egg-laying species provides unparalleled opportunities to study how sensory stimulation affects neural and cognitive development. Light stimulation during critical periods of egg incubation causes similar neural and behavioral lateralization in organisms as distant as birds and fish.^7^^,^^8^^,^^9^^,^^10^^,^^11^^,^^12^^,^^13^ Through this mechanism, environmental influences acting in specific sensitive periods have long-lasting effects on the animals’ neuroanatomical, cognitive, and social development.^6^^,^^8^^,^^12^^,^^13^^,^^14^^,^^15^^,^^16^

Conserved genetic mechanisms participate in the development of lateralization in vertebrates, including humans.^17^ In amniotes, the Nodal cascade genes cause a rightward torsion of the embryo,^18^^,^^19^ which causes asymmetric impact from sensory stimuli reaching the embryo. Under the influence of this genetic mechanism, bird embryos are placed asymmetrically in the egg,^8^^,^^13^ with the right eye facing the eggshell while the body covers the left. Exposure to light during a critical stage of egg incubation creates asymmetric stimulation of the embryos’ visual system and the subsequent emergence of functional and neuroanatomical lateralization.^7^^,^^15^^,^^20^^,^^21^^,^^22^

Since the seminal discovery that exposing chick eggs to light determines the development of cerebral and behavioral asymmetries,^7^ domestic chicks have been a central model to study these phenomena. When chicken eggs are exposed to light during incubation, light stimulation of the right eye-system causes lateralization of the thalamofugal visual pathway.^7^ This is one of the two main visual pathways in birds which provide information to the front brain.^23^^,^^24^^,^^25^ The thalamofugal pathway carries retinal projections to the thalamic nucleus opticus principalis thalami (OPT, a homolog of the mammalian lateral geniculate nucleus) in the opposite hemisphere (in birds, the optic fibers almost completely decussate at the optic chiasm.^26^^,^^27^ Although most signals from each OPT reach the ipsilateral Wulst (the main telencephalic station of this pathway), a small number of recrossing fibers also reach the contralateral Wulst.^7^^,^^25^ During incubation, light acts on the left OPT, strengthening its recrossing projections to the right Wulst and causing an anatomical asymmetry.^20^^,^^21^ This potentially increases the amount of ipsilateral visual information that reaches the right Wulst, which could thus be better equipped than the left to integrate bilateral information from the two eyes.

This light-induced anatomical asymmetry, absent in the dark-incubated chicks, has been linked to a series of behavioral differences between chicks using only the left or the right eye-system.^2^^,^^3^^,^^4^^,^^5^^,^^6^ Nevertheless, some forms of behavioral lateralization are also present in dark-incubated chicks.^28^^,^^29^ In line with that, we have shown that activation in brain areas outside the visual system can be lateralized in light- and dark-incubated chicks.^30^^,^^30^^,^^30^^,^^31^^,^^32^^,^^33^^,^^34^^,^^35^^,^^36^^,^^37^ Likewise, in a recent electrophysiological study, we have revealed functional lateralization in the response properties of bilateral and contralateral neurons in the visual Wulst of dark-incubated chicks. These spontaneous lateralization effects were further enhanced in animals hatched from light-exposed eggs.^38^

Until recently, light incubation effects and the lateralization of the tectofugal pathway, the major visual pathway to the forebrain in birds,^23^^,^^39^ was less investigated in chicks than in the thalamofugal pathway. The tectofugal visual pathway starts with projections from each retina to the contralateral optic tectum (homolog to the mammalian superior colliculus). From the tectum, visual input reaches the nucleus rotundus of the thalamus. Each tectum sends ipsilateral projections to the rotundus of the same hemisphere and recrossing projections to the rotundus of the opposite hemisphere.^40^ The rotundus then projects information from both eyes to the entopallium, the primary visual area of the avian telencephalon.^41^ Thus we can expect that the chicken entopallium, like the visual Wulst,^38^ should also contain bilaterally responsive cells. In pigeons, this pathway is lateralized, and the left entopallium has been suggested to integrate binocular information better than the right one.^42^^,^^43^ The first evidence of lateralization of the tectofugal pathway in chicks emerged in a neuroanatomy study.^20^ This work suggested the presence of an asymmetry in the proportion of ipsilateral and contralateral projections from the ventral optic tectum to the rotundus. Intriguingly, this asymmetry was not caused by light exposure of the eggs. In line with that, we showed anatomical lateralization in the entopallium of dark-incubated chicks. The right entopallium contains more parvalbumin immunoreactive neurons than the left one, potentially suggesting a dominant role of the right hemisphere in inhibitory functions without light incubation.^44^

The entopallium is the basis of many different visual functions of the avian telencephalon, such as discrimination of form, pattern, color, intensities, and motion types.^24^^,^^45^^,^^46^^,^^47^^,^^48^^,^^49^^,^^50^^,^^51^^,^^52^^,^^53^^,^^54^ In pigeons, entopallial neurons are involved in the visual working memory^41^ and show lateralized firing patterns in reward-related visual discrimination.^55^ The entopallium projects to higher associative regions of the avian visual dorsal ventricular ridge, including the mesopallium, nidopallium, and arcopallium.^39^^,^^56^^,^^57^^,^^58^ Although the entopallium is very important for visual processing in birds, this region has been primarily studied in pigeons and zebra finches. Only a few studies targeted chicken entopallium.^44^^,^^59^^,^^60^ Moreover, we did not find any published studies targeting the chicken entopallium’s functions, and to the best of our knowledge, no electrophysiological studies have been performed in chicks in this area.

Given the prominence of the tectofugal pathway in avian visual processing, we expected light incubation to affect the entopallium’s visual responses. Moreover, we expected neuroanatomical lateralization to be reflected in asymmetries of entopallial functions. The aim of the present study was thus to investigate whether light incubation impacts visual responses in the chicken entopallium. Moreover, we wanted to test whether entopallial visual responses are lateralized and what is the role of light in developing these asymmetries. We expected to observe spontaneous asymmetries already in dark-incubated chicks, which could be further modulated by light. To achieve these aims, we studied the visual response properties of entopallial neurons, using the same approach we applied to the visual Wulst.^38^ We have conducted single-unit recordings from the left and right entopallium of dark- and light-incubated chicks, following ipsi-, contra-, and bilateral visual stimulation of light flashes. To compare the strength of bilateral integration in the two hemispheres, contralaterally and bilaterally responsive units were studied separately. A previous neuroanatomical study of tecto-rotundal connectivity suggested the presence of a potential asymmetry in the proportion of ipsilateral and contralateral projections in dark-incubated chicks.^20^ We thus expected to find a light-independent asymmetry in bilateral responses, favoring the right entopallium.

## Results

**Figure 1:**
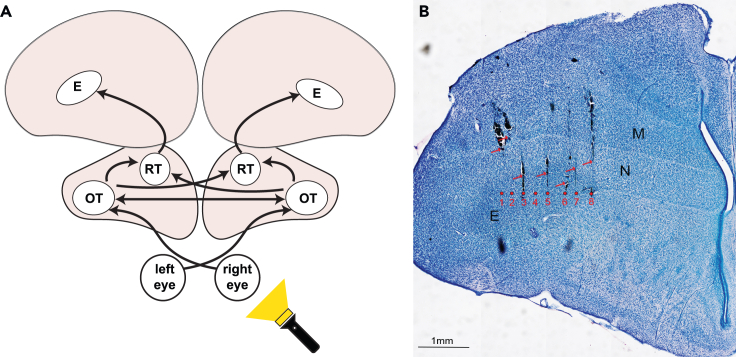
Brain region of interest (A) A schematic representation of the tectofugal pathway in birds. (B) Example of visible electrode tracks in the left hemisphere’s histological coronal brain section. Red arrows indicate visible tracks, while the red circles (numbered 1–8) indicate the expected position of the electrode tips inside the entopallium based on the deepest electrode track visible (number 8 in this case). Please note that the track of electrode 4 is not visible and has been inferred based on the tracks of other electrodes. The scale bar corresponds to 1mm. E entopallium, RT nucleus rotundus, OT optic tectum, N nidopallium, M Mesopallium.

**Figure 2:**
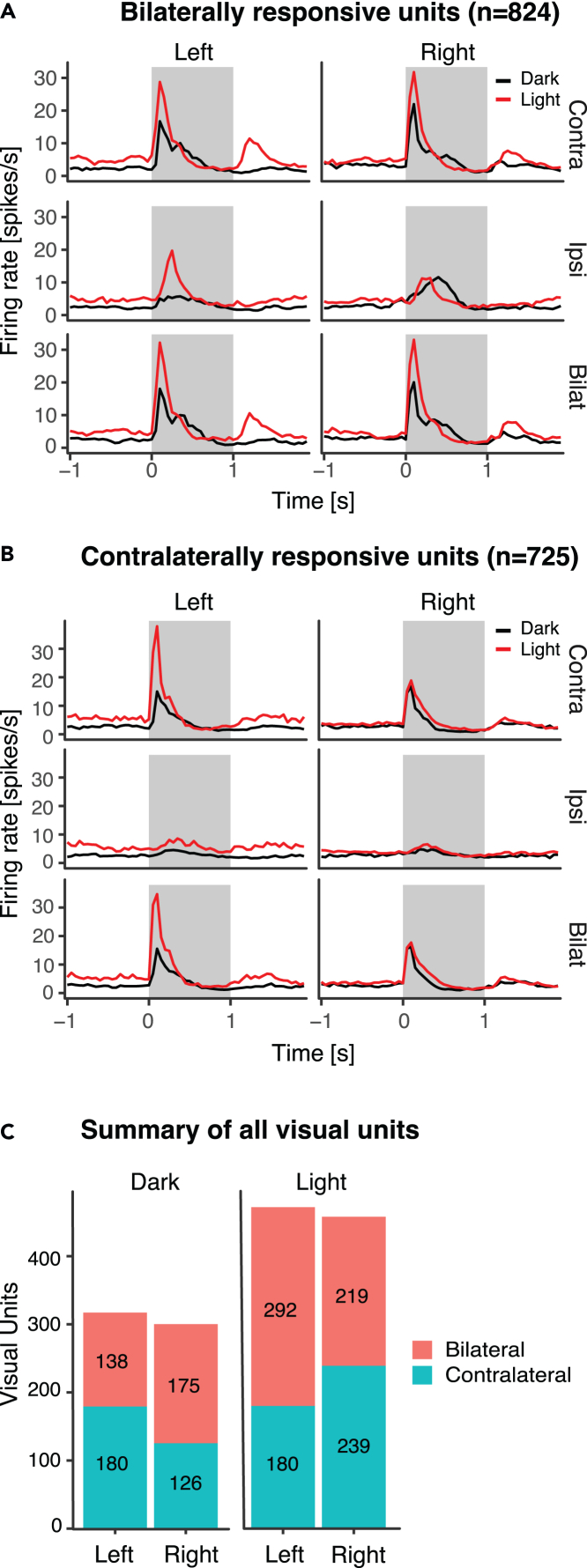
Average peristimulus time histograms showing responses to the contralateral, ipsilateral or bilateral stimulation (A) Bilaterally responsive units. (B) Contralaterally responsive units. Data from the left and right entopallium are shown separately, side by side. Black and red lines represent units recorded from dark- and light-incubated animals, respectively. (C) Summary of all visual units. The bars represent the number of bilateral (red) and contralateral (green) visual units found in each hemisphere and condition.

We successfully recorded the neural activity of 3312 units (1493 from dark-incubated and 1819 from light-incubated chicks), of which 1549 were visually responsive from the entopallium of 40 chicks (Figure 1). We classified 824 visual units (53%) as bilaterally responsive (the selection criteria for the classification are detailed in the STAR Methods). The other 725 units (47%) were almost exclusively responsive to contralateral stimulation (Figure 2). See Table 1 and Figure 2C for the distribution of visual units across different categories.

**Table 1:** Summary of visually responsive units

Incubation	Hemisphere	Number of visually responsive units	Bilateral/Contralateral response,% from visually responsive units (Number of units)
Dark	Left	318	43% (138)/57% (180)
Dark	Right	301	58% (175)/42% (126)
Dark	Left + right	619	51% (313)/49% (306)
Light	Left	472	62% (292)/38% (180)
Light	Right	458	48% (219)/52% (239)
Light	Left + right	930	55% (511)/45% (419)

Neural responses to visual stimuli were strongly affected by light incubation treatment (see PSTHs in Figure 2). The light treatment impacted the spontaneous firing rates as well as the strength and latencies of the ON and OFF responses (during 1 s during/after the stimulus presentation after subtracting the spontaneous firing rate). Furthermore, several brain hemispheric asymmetries are visible in dark- and light-incubated conditions (Figure 2). In the following, we systematically analyzed the effects of treatment (light or dark incubation) and brain hemispheric lateralization step by step in bilaterally and contralaterally responsive cells.

### Bilaterally responsive cells

**Figure 3:**
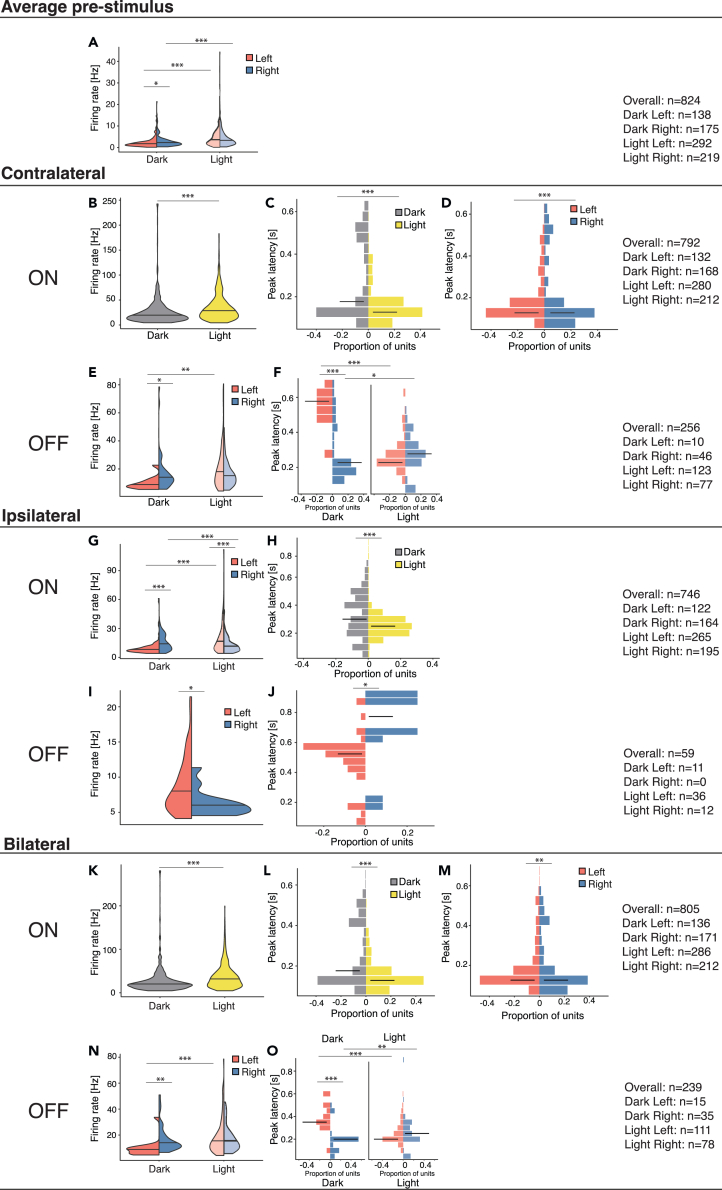
Statistically significant effects for the bilaterally responsive units Here, we plot only the statistically significant effects (for all effects, see Table 2). The violin plots (A, B, E, G, I, K, and N) represent the distribution of peak firing rates of all units (firing rates are on the y axis). If the interaction of hemispheres and treatment was significant (Table 2), the plots are split by both hemisphere and incubation conditions (A, E, G, N). For instance, in (A), it can be seen that proportionally more units with high firing rates were present in the light-incubated group of both hemispheres (violin plot on the right side of the graph). Moreover, only in the dark incubation condition, the distribution of the data are asymmetric: the plot of the left hemisphere (red) is larger at lower firing rates than the right hemisphere (blue). This indicates that the left hemisphere has proportionally more units with low firing rates. The histograms (C, D, F, H, J, L, M, and O) represent the proportion of units with different peak latencies. For instance, in (C), the light group has more units with shorter peak latencies than the dark group. The median is represented by a horizontal black stripe overlayed on each plot. Asterisks indicate significant differences as indicated by the lines (∗<0.05, ∗∗<0.01, ∗∗∗<0.001).

The light incubation significantly affected the bilaterally responsive cells at multiple levels (Figure 3; Table 2).

**Table 2:** Summary of all the results for bilaterally responsive cells

	Main results (Scheirer-Ray-Hare)		
	Treatment	Hemisphere	Interaction (Treat∗Hemi)
**Average pre-stimulus**			

Spontaneous firing rate	H_(1)_ = 65.426; p < 0.001; (η^2^ = 0.08)	H_(1)_ = 0.533; p = 0.465	H_(1)_ = 6.046; p=0.014^b^; (η^2^ = 0.08)

**Contralateral**			

ON: Peak firing rate	H_(1)_ = 41.095; p < 0.001; (η^2^ = 0.05)	H_(1)_ = 1.508; p = 0.219	H_(1)_ = 0.308; p = 0.579
ON: Peak latency	H_(1)_ = 50.536; p < 0.001; (η^2^ = 0.06)	H_(1)_ = 12.918; p < 0.001; (η^2^ = 0.01)	H_(1)_ = 1.232; p = 0.267
OFF: Peak firing rate	H_(1)_ = 3.972; p=0.046; (η^2^ = 0.02)	H_(1)_ = 0.137; p = 0.711	H_(1)_ = 5.689; p=0.017^c^; (η^2^ = 0.03)
OFF: Peak latency	H_(1)_ = 0.047; p = 0.829	H_(1)_ = 0.751; p = 0.386	H_(1)_ = 27.298; p < 0.001^d^; (η^2^ = 0.1)

**Ipsilateral**			

ON: Peak firing rate	H_(1)_ = 18.429; p < 0.001; (η^2^ = 0.02)	H_(1)_ = 0.063; p = 0.801	H_(1)_ = 61.017; p < 0.001^e^; (η^2^ = 0.1)
ON: Peak latency	H_(1)_ = 34.817; p < 0.001; (η^2^ = 0.05)	H_(1)_ = 0.811; p = 0.368	H_(1)_ = 0.591; p = 0.442
OFF: Peak firing rate	H_(1)_ = 1.214; p = 0.271	H_(1)_ = 4.895; p=0.027; (η^2^ = 0.05)	H_(1)_ = 52.764; p = 0.598
OFF: Peak latency	H_(1)_ = 0.868; p = 0.352	H_(1)_ = 9.53; p=0.002; (η^2^ = 0.19)	H_(1)_ = 45.478; p = 0.841

**Bilateral**			

ON: Peak firing rate	H_(1)_ = 49.862; p < 0.001; (η^2^ = 0.06)	H_(1)_ = 0; p = 0.986	H_(1)_ = 0.194; p = 0.659
ON: Peak latency	H_(1)_ = 65.849; p < 0.001; (η^2^ = 0.07)	H_(1)_ = 10.026; p=0.002; (η^2^ < 0.01)	H_(1)_ = 0.903; p = 0.342
OFF: Peak firing rate	H_(1)_ = 5.580; p = 0.018; (η^2^ = 0.01)	H_(1)_ = 2.478; p = 0.115	H_(1)_ = 7.376; p=0.007^f^; (η^2^ = 0.05)
OFF: Peak latency	H_(1)_ = 0.004; p = 0.948	H_(1)_ = 4.452; p=0.035; (η^2^ = 0.02)	H_(1)_ = 25.674; p < 0.001^g^; (η^2^ = 0.12)

**Post-hoc (Dunn, 1964)**			

Dark: Left vs. Right	Light: Left vs. Right	Left: Dark vs. Light	Right: Dark vs. Light
^b^ z = −2.3849; p < 0.018	z = 0.9443; p = 0.345	z = −7.4208; p < 0.001^a^	z = −4.0502; p < 0.001^a^
^c^ z = −2.0593; p=0.039	z = 1.2591; p = 0.208	z = −3.0577; p=0.002^a^	z = −0.5580; p = 0.577
^d^ z = 5.1564; p < 0.001^a^	z = −1.2087; p = 0.227	z = 4.652; p < 0.001^a^	z = −2.3880; p=0.017
^e^ z = 6.2878; p < 0.001^a^	z = 4.6416; p < 0.001^a^	z = −8.5978; p < 0.001^a^	z = 2.3502; p=0.019
^f^ z = −3.1293; p=0.002^a^	z = −0.2470; p = 0.805	z = −3.5881; p < 0.001^a^	z = −0.2843; p = 0.776
^g^ z = 5.4814; p < 0.001^a^	z = −0.2848 p = 0.776	z = 4.1124; p < 0.001^a^	z = −2.9609; p=0.003^a^

Effect size η^2^: 0.01- < 0.06 (small effect), 0.06 - < 0.14 (moderate effect) and ≥ 0.14 (large effect).

In case of significant interaction, post-hoc analyses were performed, which are reported in the lower section of the table. Each significant interaction and corresponding post-hoc is labeled with a superscript letter (b-g).

a significant also after a Bonferroni adjustment for multiple comparisons.

#### Spontaneous firing rates

Interaction of treatment and hemispheres was present in the spontaneous firing rates of the pre-stimulus intervals (Table 2b). Overall, in both hemispheres, more neurons showed higher spontaneous firing rates in the light-incubated chicks than those from dark incubators (in Figure 3A). However, post hoc analysis revealed that in the dark-incubated chicks, proportionally more neurons with lower spontaneous firing rates were present in the left than in the right hemispheres (Figure 3A). Such a brain hemispheric spontaneous firing rate asymmetry was absent in light-incubated chicks.

#### Responses to contralateral stimulation

In light-incubated chicks, more units showed stronger and faster ON responses to contralateral stimulation than dark-incubated chicks, regardless of the brain hemisphere (Figures 3B and 3C). At the same time, independent of the incubation condition, more units of the right hemisphere showed faster contralateral ON responses than the left hemisphere (Figure 3D). Light incubation also affected the OFF responses to contralateral stimulation. However, OFF responses showed significant interactions between treatment and hemispheres (Table 2; Figures 3E and 3F). In dark-incubated chicks, stronger and faster OFF responses were present in the right hemisphere, but such asymmetry was absent in light-incubated chicks. Moreover, in the left hemisphere only, light incubation induced stronger responses than dark-incubated chicks. Light incubation had opposite effects on the latency of OFF responses in the two hemispheres: while in the left hemisphere, the light treatment induced faster responses, in the right hemisphere faster responses were observed in dark-incubated chicks.

#### Responses to ipsilateral stimulation

A significant interaction between treatment and hemispheres emerged for the peak firing rates for the ipsilateral ON responses (Table 2). In dark-incubated chicks, stronger responses were recorded more often in the right than in the left hemisphere. In contrast, stronger peaks were more abundant in the left than the right hemisphere in the light-incubated chicks. Thus, light incubation inverted the asymmetry of ipsilateral ON responses. Moreover, light also had opposite effects on the two hemispheres: while in the left hemisphere, light incubation induced stronger ON responses, the opposite was true in the right hemisphere (Figure 3G). In both brain hemispheres, light also induced faster ON responses (Figure 3H). Moreover, regardless of the treatment condition, stronger and faster OFF responses to ipsilateral stimulation were present in the left hemisphere (Figures 3I and 3J).

#### Responses to bilateral stimulation

Light incubation affected bilateral ON responses. Regardless of the hemisphere, stronger and faster responses (Figures 3K and 3I) were present in the light incubation condition. At the same time, independent of the treatment, faster responses were present in the right hemisphere (Figure 3M). For the bilateral OFF responses, light incubation significantly interacted with the hemisphere (Table 2). Post hoc analysis showed a brain-hemispheric asymmetry in the dark-incubated chicks, which was absent in the light-incubated ones. In dark-incubated chicks only, the right hemisphere had stronger and faster responses than the left one. Moreover, in the left hemisphere only, light incubation caused the emergence of stronger responses (Figure 3N). Finally, light stimulation had the opposite effect on response latencies in the two hemispheres: while in the left hemisphere light incubation caused faster responses, the opposite was true in the right hemisphere (Figure 3O).

#### Excitatory and inhibitory integration of responses to bilateral stimulation

**Figure 4:**
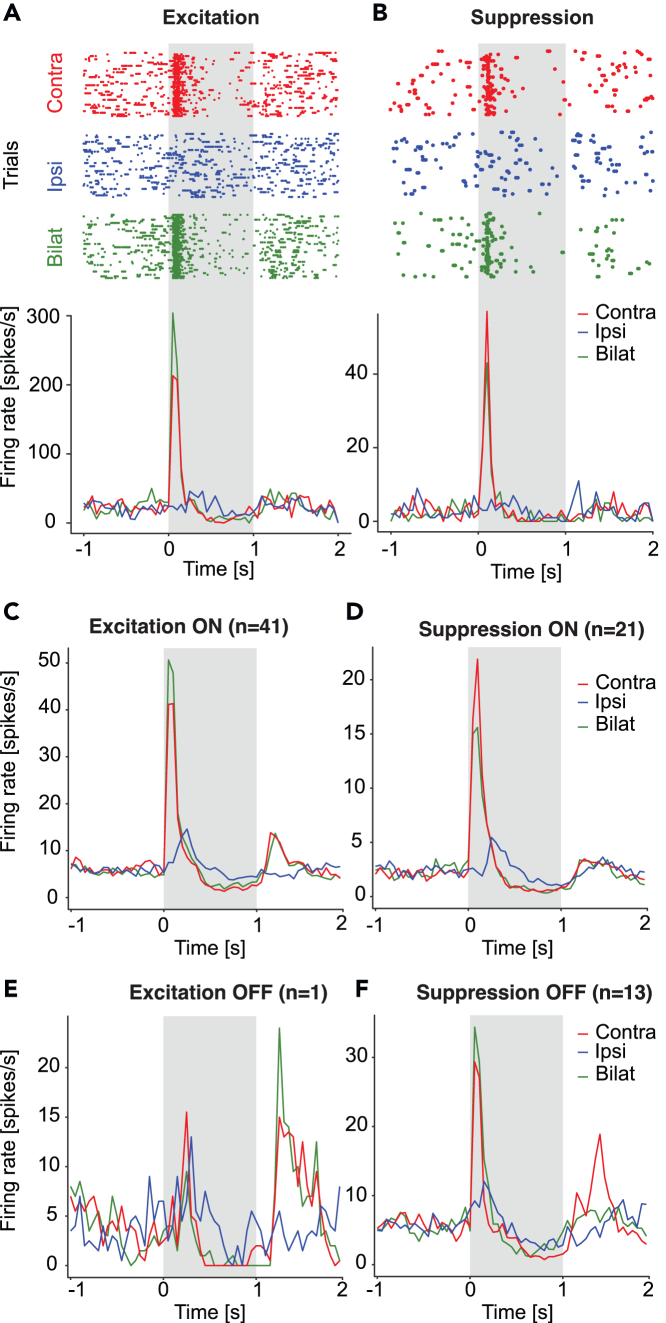
Excitation and suppression by the simultaneous visual stimulation of both eyes (A) An example of a unit showing excitatory interaction (raster plots on the top and peristimulus time histograms in the lower part). In this case, the response to bilateral stimulation (green) is higher than to contralateral stimulation (red). (B) An example of a unit reflecting suppression by stimulation of the two eyes, where the response to the bilateral stimulation is lower than to contralateral stimulation. (C–F) represent the average PSTHs of all excitation ON, suppression ON, excitation OFF and suppression OFF units, respectively.

Significantly higher responses to bilateral stimulation than contralateral stimulation were present in 41 ON units, indicating excitatory interaction when both eyes are stimulated (Figure 4C). Significantly lower peak response when both eyes were stimulated than contralateral stimulation was present in 21 units, indicating suppression (Figure 4D). Examples of two units with excitatory and suppressed responses to bilateral stimulation are shown in Figures 4A and 4B, respectively. Moreover, we isolated one OFF unit with an excitatory integration (Figure 4E) and 13 OFF units with an inhibitory integration (Figure 4F).

### Contralaterally responsive cells

**Figure 5:**
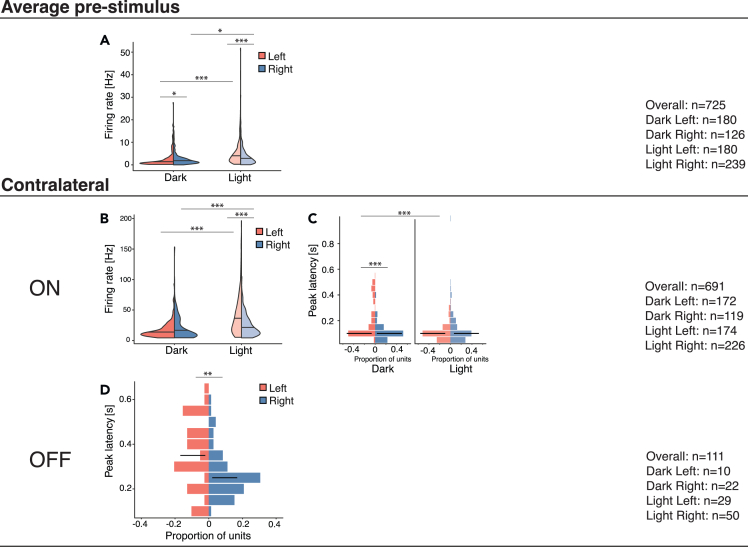
Statistically significant effects for the contralaterally responsive units The violin plots (A and B) represent the distribution of peak firing rates of all units (firing rates are on the y-axis). Since the interaction of hemisphere and treatment was significant (Table 3), the plots are split by both hemisphere and incubation. The histograms (C and D) represent the proportion of units with different peak latencies. The median is represented by a horizontal black stripe on each plot. Asterisks indicate significant differences as indicated by the lines (∗<0.05, ∗∗<0.01, ∗∗∗<0.001).

The light incubation also affected the contralateral responsive units. In both hemispheres, light-incubated chicks had higher spontaneous firing rates than dark-incubated chicks. The spontaneous firing rate also showed a significant interaction between treatment and hemispheres (Table 3; Figure 5A). In dark-incubated chicks, a higher spontaneous firing rate was found in the right hemisphere, while in light-incubated chicks, this asymmetry was reversed.

**Table 3:** Summary of the results for contralaterally responsive cells

	Main results (Scheirer-Ray-Hare)		
	Treatment	Hemisphere	Interaction (Treat∗Hemi)
**Average pre-stimulus**			

Spontaneous firing rate	H_(1)_ = 58.782; p < 0.001; (η^2^ = 0.08)	H_(1)_ = 2.227; p = 0.136	H_(1)_ = 17.681; p < 0.001^b^; (η^2^ = 0.1)

**Contralateral**			

ON: Peak firing rate	H_(1)_ = 77.287; p < 0.001; (η^2^ = 0.1)	H_(1)_ = 13.781; p < 0.001; (η^2^ = 0.01)	H_(1)_ = 10.892; p=0.001^c^; (η^2^ = 0.13)
ON: Peak latency	H_(1)_ = 20.874; p < 0.001; (η^2^ = 0.04)	H_(1)_ = 7.322; p=0.007; (η^2^ = 0.02)	H_(1)_ = 28.859; p < 0.001^d^; (η^2^ = 0.09)
OFF: Peak firing rate	H_(1)_ = 3.448; p = 0.063; (η^2^ = 0.02)	H_(1)_ = 0.861; p = 0.353	H_(1)_ = 2.156; p = 0.142
OFF: Peak latency	H_(1)_ = 0.245; p = 0.621	H_(1)_ = 9.889; p=0.002; (η^2^ = 0.08)	H_(1)_ = 2.519; p = 0.113

**Post hoc (Dunn, 1964)**			

Dark: Left vs. Right	Light: Left vs. Right	Left: Dark vs. Light	Right: Dark vs. Light
^b^ z = −2.2382; p=0.025	z = 3.8599; p < 0.001^a^	z = −8.4459; p < 0.001^a^	z = −2.2650; p=0.024
^c^ z = −0.1224; p = 0.903	z = 4.9657; p < 0.001^a^	z = −8.6481; p < 0.001^a^	z = −3.6592; p < 0.001^a^
^d^ z = 5.8490; p < 0.001^a^	z = −1.4033; p = 0.161	Z = 7.0121; p < 0.001^a^	z = −0.7504; p = 0.453

η^2^: 0.01- < 0.06 (small effect), 0.06 - < 0.14 (moderate effect) and ≥ 0.14 (large effect).

Each significant interaction and corresponding post-hoc is labeled with a superscript letter (b-d).

a significant also after a Bonferroni adjustment for multiple comparisons.

ON responses, too, showed significant interactions between treatment and hemispheres (Table 3). In both hemispheres, the light incubation induced stronger responses. Moreover, light exposure induced an asymmetry that was absent in dark-incubated chicks: for light-incubated chicks only, stronger ON responses were present in the left than in the right hemisphere (Figure 5B). Response latencies, however, revealed a different pattern of results (Table 3). Dark-incubated chicks showed faster responses in the right hemisphere compared to the left. An asymmetry which is absent in light-incubated animals. This could stem from the fact that light incubation selectively affected responses in the left hemisphere by making them faster, abolishing the asymmetry (Figure 5C). Finally, regardless of the light incubation, we observed faster OFF responses in the right hemisphere (Figure 5D).

## Discussion

### The effect of light on the general responsiveness of visual units

Light incubation profoundly affected the entopallium’s visual responses, inducing stronger and/or faster responses. We previously observed a similar effect of light on visual responses in the visual Wulst.^38^ This suggests a similar action of light on the maturation of visual responses in the tectofugal and thalamofugal visual pathways. In both cases, light exposure seems to have increased the baseline activity, the sensitivity to changes in visual stimulation and the diversity of neural responses. This is important since, until recently, the behavioral effects of in-egg light exposure have been interpreted as stemming only from changes in the lateralization profile.^5^^,^^61^ Future studies should thus aim to disentangle cases in which the effects of light incubation on behavioral performance are due to increased lateralization of the visual pathways from cases in which light acts by stimulating general visual responsiveness in a non-lateralized fashion.

Effects of light on general visual responsiveness either appeared to be identical in both hemispheres (henceforth hemisphere-independent effects, for simplicity) or affected one hemisphere only. Importantly, light-induced changes affecting one hemisphere only do not automatically cause the emergence of functional lateralization. For example, if light acts upon a pre-existing asymmetry, it can either enhance it or counteract it, thus equalizing the response profiles of the two hemispheres. Functional lateralization in dark- and light-incubated animals will thus be discussed in a separate section.

Hemisphere-independent effects include the increase in spontaneous firing rates; in bilaterally responsive units, the presence of stronger and faster ON responses to contralateral and bilateral stimulation and of faster ipsilateral ON responses; in contralaterally responsive units, stronger ON responses. Cases in which light exposure selectively affected the left entopallium included, for bilateral units, stronger contralateral and bilateral OFF responses, and, for contralateral units, faster ON responses. Finally, for bilateral cells we could observe instances in which light exposure affected both hemispheres, but in opposite directions. While in the left entopallium, light exposure induced faster contralateral and bilateral OFF responses and stronger ipsilateral ON responses, in the right entopallium slower and weaker responses were observed in light-incubated chicks (compared to dark-incubated ones). Overall, light exposure seemed thus to increase entopallial circuits’ ability to rapidly detect changes in a visual scene (i.e., to produce strong and fast responses to both the appearance of the visual stimuli and their offset). This is well illustrated in Figure 2A: light-incubated chicks not only have higher peaks at the onset of the visual stimuli but also present OFF responses, which are virtually absent in dark-incubated animals.

**Figure 6:**
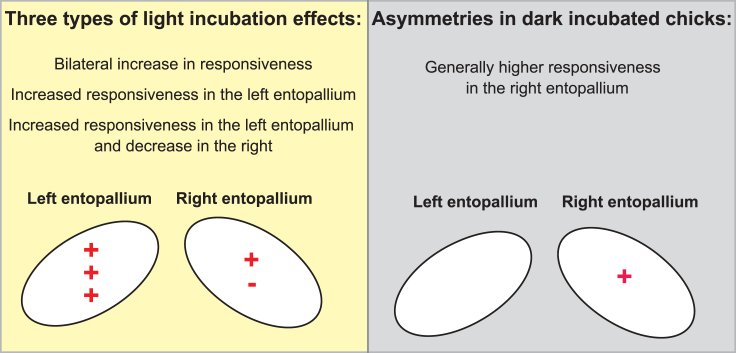
Summary of the main findings

When we consider all these light-induced effects, a common pattern emerges: light exposure either increased the responsiveness of visual units in both the left and the right entopallium, or it selectively facilitated the responses in the left entopallium, or finally, it simultaneously facilitated the responsiveness in the left entopallium and hindered responsiveness in the right one (Figure 6).

The facilitating effect of light on the left entopallium is in line with the fact that in-Ovo light exposure should cause stronger visual stimulation to the left telencephalon due to the total decussation of fibers at the optic chiasm. In contrast, the hemisphere-independent effects of light can be explained by the fact that the tectofugal visual pathway presents recrossing tecto-rotundal projections.^40^ This allows the inputs caused by light on the right eye to reach both the left and the right entopallium. We believe that tectofugal recrossing projections may have an important role in these hemisphere-independent effects of light. In fact, we observed a high prevalence of bilaterally responsive units in the entopallium (about half of the recorded units appeared to integrate information from both eyes, while the other units responded only to contralateral stimulation). Entopallial responses are still predominantly driven by stimulation of the contralateral eye since even bilaterally responsive units still respond stronger to contralateral than to ipsilateral stimuli. However, the proportion of bilateral units we observed here (824 out of 1549, 53%) is substantially higher (z = −12.52, p < 0.01) than in the visual Wulst (478 out of 1544, 31%).^38^ Integrating information from the two eyes is thus a prominent feature of visual processing in the entopallium, even more than in the visual Wulst.

In birds with laterally placed eyes, an important function of bilateral integration was proposed to be the inhibition of competing information from the non-fixating eye.^62^^,^^63^^,^^64^ Following this hypothesis, we expected to observe that bilateral stimulation would inhibit or suppress the neural response compared to purely contralateral stimuli. In Wulst, however, about the same number of increased or suppressed responses were found to bilateral stimulation, which already seemed to conflict with the idea of inhibitory processing as the main function of bilateral integration.^38^ Furthermore, in entopallium, we found a very high prevalence of excitatory ON responses to bilateral stimulation (increased ON responses were two times more frequent than suppressed ones), marking a difference in bilateral responses between the thalamofugal and the tectofugal recipients. This could reflect the excitatory/inhibitory functions of recrossing projections in the two pathways. In contrast, bilateral OFF responses were almost exclusively suppressive in entopallium, like in the Wulst. However, these were not very frequent overall. Thus, our results do not seem to support the idea that bilateral integration has a predominantly inhibitory function. Indeed, hemispheres have to be able to switch between inhibitory modes (under conditions of hemispheric competition) and interhemispheric synchronization (during cooperation for shared computation). As proposed for pigeons^65^ such a function could be supported via interactions of the commissura anterior in birds. However, the role of inhibitory and excitatory networks in mediating the effects of light on chicks’ visual pathways is still under-investigated in chicks.

### The lateralization of visual responses in dark-incubated and light-incubated chicks

In many instances, we could observe that entopallial visual responses were asymmetric already in the dark-incubated chicks. Both bilaterally and contralaterally responsive units show higher baseline activity in the right hemisphere of dark-incubated chicks. Moreover, the right entopallium showed facilitation for responses to contralateral or bilateral stimulation, compared to the left entopallium (e.g., stronger and faster responses to the offset of contralateral stimulation; in bilateral units only, faster responses to the onset of contralateral stimulation; faster and stronger responses to the offset of bilateral stimulation and faster responses to its onset). Stronger responses to the onset of ipsilateral stimulation were also observed in the right hemisphere, compared to the left one. The only exception to this general trend for a spontaneous right-hemispheric dominance was that responses to the offset of ipsilateral stimulation were faster and stronger in the left entopallium.

Overall, the frequent presence of faster responses in the right entopallium is in line with what was observed in the right visual Wulst of dark-incubated chicks.^38^ Faster visual responses in the right hemisphere are consistent with the proposed specialization of the left eye-system for “ready response to releaser” functions.^12^ According to this theory, visual projections to the right hemisphere support fast responses to releasers of species-specific behaviors and to novel stimuli. This is believed to be at the basis of the left eye-system dominance for monitoring biologically relevant stimuli, like predators and conspecifics (e.g., see^4^^,^^61^).

The presence of functional asymmetries in chicks that were not exposed to light during incubation is overall in line with what we observed in the visual Wulst.^38^ This shows that, also within the tectofugal pathway, lateralization in neural response properties can develop without asymmetric light stimulation. Overall, our results in the visual Wulst and in the entopallium undermine the original belief that visual lateralization in birds emerges only as a consequence of the asymmetric exposure to light. These results are, however, consistent with the accumulating evidence of behavioral asymmetries (e.g.,^28^^,^^29^^,^^66^^,^^67^^,^^68^^,^^69^) and lateralized expression of brain activity/plasticity markers in the brains of dark-incubated chicks (e.g.,^30^^,^^30^^,^^30^^,^^31^^,^^32^^,^^33^^,^^34^^,^^35^^,^^36^^,^^37^). Anatomical lateralization has also been found within the entopallium of dark-incubated chicks, albeit with a small effect size.^44^ In the study,^44^ the right entopallium presented a higher density of parvalbumin-expressing neurons. In mammals, these are a type of GABAergic inhibitory neurons, suggesting rightward lateralization of inhibitory functions in the entopallium. However, at the current state of knowledge, we are not able to directly connect the anatomical results with our electrophysiological findings.

Most of the asymmetries observed in dark-incubated chicks were not affected by light exposure, developing in both treatment groups. However, light exposure suppressed some of the asymmetries present in the dark-incubated groups, which were absent in light-incubated animals. Only in dark-incubated chicks, bilateral units had stronger and faster OFF responses to contralateral or bilateral stimulation in the right entopallium than in the left. Likewise, contralateral units showed faster ON responses in the right than in the left entopallium of dark-incubated chicks only. This could be due to the stronger light stimulation received by the left entopallium, compared to the right entopallium, thanks to the light reaching the right eye. Moreover, the asymmetric light stimulation of the two eyes reversed the direction of some spontaneous asymmetries present in dark-incubated animals. In contrast to what we observed in dark-incubated animals, contralateral units of light-exposed chicks showed higher spontaneous activity in the left entopallium. Likewise, light-incubated animals presented stronger responses to the onset of ipsilateral stimulation in the left entopallium. Finally, light incubation also induced two *de novo* asymmetries that were absent in dark-incubated animals. Only in light-incubated chicks bilateral units showed stronger responses in the left than in the right hemisphere to the offset of contralateral stimulation; likewise, only in light-incubated chicks contralateral units showed stronger responses to the onset of visual stimulation in the left than in the right hemisphere. Overall, in line with what can be expected based on the structure of the avian visual pathways, light exposure increased the responsiveness of the left entopallium (to which the light-exposed right eye mostly projects). When this effect acted upon the right-hemispheric advantage often present in dark-incubated chicks, light exposure either abolished or reversed the asymmetry. In line with that, bilaterally responsive units were more abundant in the right hemisphere of dark-incubated animals and in the left hemisphere of light-incubated animals.

This picture is consistent with what we know from pigeons, in which light exposure elicits lateralization of the tectofugal pathway, causing stronger binocular integration in the left hemisphere.^15^^,^^42^ In the pigeons, light increases soma size in the neurons of superficial layers of the left tectum, while decreasing it for the neurons in the deeper layers.^70^ This is believed to cause a reduction of the recrossing projections from the left tectum to the right rotundus. At the same time, recrossing projections from the right tectum to the left rotundus remain unaffected, conferring a potential advantage to the structures of the left tectofugal pathway in terms of binocular integration.^42^ In contrast, studies on chicks could not identify such a clear asymmetry in the tectofugal projections.^20^ Indeed, until now evidence of tectofugal lateralization in chicks was scarce,^44^^,^^71^ while prominent light-dependent asymmetries were found in chicks’ thalamofugal projections.^20^^,^^72^ This has been viewed as evidence of a dichotomy between the two species. A similar behavioral lateralization profile would thus emerge from two distinct sets of neural mechanisms in chicks and pigeons (but see in the study by Manns et al. ^15^) for an alternative on the different contributions of the two hemispheres in chicks’ and pigeons’ behavior). Here, we provide clear evidence of light-dependent lateralization in the tectofugal pathway of domestic chicks, indicating that similar mechanisms might actually be at play in both species. Our study confirms that the phenomena observed in these two models can be generalized behind the confines of species-specific idiosyncratic effects, informing a wide range of future studies. While differences between species are undeniable, we show that in both pigeons and chicks, light incubation causes similar lateralization of the tectofugal visual pathway. However, until now, neither lateralization of the thalamofugal projections nor light-independent lateralization has been reported in the pigeon brain (but see in the study by Letzner et al. ^73^ or indirect behavioral evidence). Future studies should tackle this issue using comparable approaches in the two species. This could be done by taking advantage of the simple but powerful design we implemented here and in our corresponding paper on visual Wulst.^38^

### Limitations of the study

Last but not the least, we would like to point out some limitations of our study, mostly regarding the recording location. Using an electrode array that covered 1.75mm in the medial-lateral orientation, we sampled a relatively large area. However, our array had only 16 recording sites with 0.25mm spacing. This prevents us from having a more detailed picture of the internal functional organization of the entopallium. A similar concern is that entopallium is a large structure within the telencephalon of chicks. The stereotactic coordinates we used targeted the intermediate portion of the entopallium, both along the anteroposterior axis and the ventrodorsal axis. Our results are thus representative of the properties of this entopallial subregion only. Other portions of the entopallium may present different response patterns. Previous research in pigeons has shown that lesions to different anteroposterior regions of the entopallium can result in deficits in different visual discrimination tasks.^49^ Functional subdivisions are likely to be present also in the chick entopallium.^74^ This has also been suggested by our neuroanatomical study, which showed regional differences in the distribution of parvalbumin immunoreactive cells within the entopallium of domestic chicks.^44^ Moreover entopallium is at the basis of many visual functions which we did not target with our stimuli. For instance, neurons in the entopallium are sensitive to different kinds of motions^75^^,^^76^ and several static visual properties (for Reviews, see^25^^,^^39^). While our stimuli were chosen to elicit a large number of visual responses, we did not study populations of neurons with more specific response properties. Future studies should thus systematically target different entopallial coordinates, using varied stimuli to gain further information on the presence and nature of functional subdivisions within the entopallium. In this study, we follow the classical approach to the study of the primary sensory visual process by recording from anesthetized animals. This has the advantage of reducing the impact of higher-order processes on visual responses and allows us to obtain highly controlled data from an exceptionally large number of animals compared to most electrophysiological studies. However, this approach also has potential limitations since the use of anesthetics affects the neural responses compared to awake animals (e.g.,^77^). Follow-up studies should thus test how light incubation affects the lateralization of awake animals. Finally, even though we used animals of both sexes, we did not test for sex effects, which was beyond the aims of the study. This aspect can be targeted in future research based on specific *a priori* hypotheses on the role of sex.

## STAR★Methods

### Key resources table

**Table:** 

REAGENT or RESOURCE	SOURCE	IDENTIFIER
**Chemicals, peptides, and recombinant proteins**		

Giemsa dye	Sigma-Aldrich, St. Louis, USA	Cat# MG500
Eukitt® Quick-hardening mounting medium	Sigma-Aldrich, St. Louis, USA	Cat# 03989

**Deposited data**		

Raw and analyzed data	This manuscript	https://doi.org/10.5281/zenodo.10651130

**Experimental models: Organisms/strains**		

*Gallus gallus domesticus,* Aviagen ROSS 308 strain	CRESCENTI Società Agricola S.r.l. –AllevamentoTrepola– cod. Allevamento127BS105/2	N/A

**Software and algorithms**		

R Studio	R Studio Team^78^	URL http://www.rstudio.com/
R Code used for data analysis	This manuscript	https://doi.org/10.5281/zenodo.10651130
Neuroexplorer (v. 5)	Neroexplorer, Colorado Springs, CO, 80906, USA	www.neuroexplorer.com
Offline Sorter (OFS) software (v3.3.5)	Plexon Inc, Dallas, Texas 75206, USA	https://plexon.com/
PsychoPy-tool (v3.0)	Peirce et al.^79^	https://psychopy.org/

**Other**		

16 channels platinum-iridium microelectrode array	MicroProbes for life science, USA	Cat# MEA-PI-A-3-00-16-2.0-5-3-250-250-1-1SS-1

### Resource availability

#### Lead contact

Further information and requests for resources and reagents should be directed to and will be fulfilled by the Lead Contact, Uwe Mayer (uwe.mayer@unitn.it).

#### Materials availability

This study did not generate new, unique materials.

#### Data and code availability

• Data have been deposited at Zenodo and are publicly available as of the date of publication. The DOI is listed in the key resources table.
• All original code has been deposited at Zenodo and is publicly available as of the date of publication. DOIs are listed in the key resources table.
• Any additional information required to reanalyse the data reported in this paper is available from the lead contact upon request.

### Experimental model and study participant details

We used forty laboratory-hatched domestic chicks (*Gallus gallus domesticus*) of the Aviagen ROSS 308 strain. The fertilised eggs were obtained from a local commercial hatchery (CRESCENTI Società Agricola S.r.l. –AllevamentoTrepola– cod. Allevamento127BS105/2) and incubated at 37.7°C, with a humidity of 60% in standard incubators (Marans P140TU-P210TU).

The eggs were divided into two experimental groups: ‘Dark Incubated’ (N = 20, of which 10 males) were in darkness from embryonic days E0 to E21. ‘Light Incubated’ (N = 20, of which 10 males) were light stimulated during the sensitive period (Rogers 1982) from the morning of day E18 to the evening of E19. For light stimulation, we used 15 LEDs (270 lm) attached to a plastic panel (38 x 38 cm^2^) placed on the ceiling of the incubator. The light intensity at the level of the eggs was 1036 lx. Chicks hatched in dark incubators. They were then housed for four days individually in metal cages (28 cm wide × 32 cm high × 40 cm deep) at a constant room temperature of 30–32°C and a 14 h light and 10 h dark regime with food and water available *ad libitum*. The electrophysiological recordings were performed on post-hatching day four.

All experiments were carried out following and complied with the ethical guidelines current to European and Italian laws and the ethical standards of the University of Trento. All the experiments and the experimental procedures were licensed by the ‘Ministero della Salute, Dipartimento Alimenti, Nutrizione e Sanità Pubblica Veterinaria’ (permit number 1061/2016-PR).

### Method details

#### Neural recording

The recordings were performed in fully anaesthetised chicks. For this purpose, the birds were food deprived for 2 hours. They were then administered 0.7 ml urethane solution (20% urethane in 0.9% NaCl) in three steps of intramuscular injections (0.4 ml, 0.2 ml, 0.1 ml) with 30 minutes of time intervals between each step. When the bird became unresponsive, the head was fixed (with the beak oriented horizontally) in a stereotaxic head holder.^80^ The feathers were removed with wax stripes, and local anaesthetic (2.5% lidocaine gel, AstraZeneca S.p.A.) was applied to the head’s skin before exposing the skull. Craniotomy was performed above the entopallium of both hemispheres, and the meninges were removed. The eyelids of both eyes were opened and fixed with adhesive tape. The nictitating membrane (fully functional in the anaesthetised birds) protected the eye from drying during recordings. The stereotactic holder with the bird was placed in the experimental setup consisting of an anti-vibration table (Thorlab Nexus, 110x95 cm) with lateral walls and a computer monitor in front of the chick. The outer sides of the lateral walls were covered with aluminium foil and grounded for shielding. Also, the table’s surface and the chick’s skin were grounded. The recording was performed in a dim light-illuminated room.

Short flashing stimuli were presented randomly to the left, the right or both eyes through two fibre-optic cables (Carl Zeiss). Each flashing stimulus was presented for 1 sec, followed by an inter-stimulus interval (ISI) of 4 sec and repeated 40 times. The two fibre-optic cables were placed laterally at a 2mm distance from the eyes. The other ends of the two fibre-optic cables were placed directly over the surface of a computer monitor (AOC AGON AG271QX; LCD, size: 27 inches; resolution: 2560 x 1440 pixels; refresh rate: 144Hz; response time: 1ms). The flashing stimuli were two white circles appearing randomly one by one or together on the computer monitor directly beneath the two fibre-optic cables. The frontal visual field of the birds was additionally occluded by black cardboard preventing them from seeing the stimuli on the computer monitors in front. The stimuli were created using the PsychoPy-tool (v3.0)^79^ based on Python. The timing of stimuli presentations was detected by photodiodes attached to the computer monitor outside the lateral walls of the setup.

For recording, we used a 16 channels platinum-iridium microelectrode array (impedance: 2.0 Megohms, MicroProbes for life science, USA). The electrode array had a 2x8 arrangement with a distance of 250μm between each electrode. It covered a region of 1.75 mm in the medial-lateral orientation and 250μm in the anteroposterior orientation of the brain. The bregma was used as a 0.0 coordinate to estimate the brain coordinates for electrode placement. With a motorised micro manipulator, the electrode array was placed at the following coordinates: anterior (A) 3.0 mm to A 3.25 mm and lateral (L) 4.0 mm to L 5.75 mm for the right hemisphere or L -4.0 mm to L -5.75 mm for the left hemisphere. The electrode array was then slowly lowered into the brain at an angle of 45° degrees to the depths coordinate of D 2.7 mm as estimated from the brain’s surface.

Data was acquired by the Plexon multichannel system (Plexon, Dallas, USA). The signals were pre-amplified (20x) with a 16ch head stage (Model number: PX.HST/16V-G20-LN). The signals were then amplified 1000×, digitalised and filtered (300 Hz high-pass filter, 3 kHz low-pass filter and 50Hz noise removal). The averaged signal across channels method for referencing (CAR-Common Average Referencing) of the PlexControl system provided a good signal-to-noise ratio. Spikes were detected online with the PlexControl software. The automatic thresholding was set at 4-sigma from the noise level average. The software ‘Neuroexplorer’ (v.5.) was synchronously running with the PlexControl software during recording. This allowed us to observe the peristimulus time histograms (PSTH) and raster plots of individual units already during recording.

#### Histology

Chicks were overdosed with ketamine/xylazine solution (1:1 ketamine 10 mg/ml + xylazine 2 mg/ml) and perfused with phosphate-buffered saline (PBS; 0.1 mol, pH = 7.4, 0.9% sodium chloride, 5°C) and 4% paraformaldehyde (PFA). After post-fixation of the brains for two days in 20% sucrose in solution and two days in 30% Sucrose in PFA solution, coronal sections (60 μm) were cut using a cryostat (Leica CM1850 UV). The sections were mounted on glass slides, stained with Giemsa dye (MG500, Sigma-Aldrich, St. Louis, USA) and cover-slipped with Eukitt (FLUKA). Electrode tracks in histological sections were examined with a Zeiss stereomicroscope (Stemi 508, Carl Zeiss, Oberkochen, Germany). An example is shown in Figure 1.

### Quantification and statistical analysis

#### Data analysis

Neural spikes were sorted with the Offline Sorter (OFS) software (v3.3.5., Plexon Inc). We used the ‘K-Means scan’ method to automatically separate two-dimensional principal component clusters into units across all channels. The timestamps were exported through Neuroexplorer to Matlab (R2018a). All further analyses were based on each unit’s PSTH (bin size 25ms). Only strongly visually responsive units were considered for further analysis. The selection criterion was that in at least one of the presentation conditions (ipsilateral, contralateral and bilateral presentation), the peak firing rate during the 1 sec of stimulus presentation (ON response) or during the 1 sec after stimulus presentation (OFF response) had to be at least two-fold higher than any peak occurring in the 1 sec before stimulus presentation. Units with a peak firing rate lower than 5 Hz were excluded from further analyses.

We used the selection steps described in the following paragraph to separate units showing any indication of bilateral integration from the population of units almost exclusively responding to contralateral eye stimulation (Figure 2). The selection was done step by step, based on the features indicative of bilateral integration and applied to both ON responses (during 1 sec stimulus presentation) and OFF responses (during 1 sec after stimulus presentation). Thus, each unit had to satisfy at least one of the following criteria to be considered bilaterally responsive.

All cells responded to contralateral stimulation. Thus as the first indication of bilateral integration, we selected units that showed an additional response to ipsilateral eye stimulation. The ipsilateral peak response had to be four standard deviations (sigma) above the mean noise level. In the second step, we selected units whose peak response to bilateral stimulation differed from the contralateral one. The peaks were compared with a Wilcoxon rank sum test with continuity correction (p<0.05), indicating that the contralateral response was modulated in the presence of concurrent ipsilateral stimulation. The same test was used in the third step. Here, instead of the peaks, we compared the firing rates during 1 sec of stimulus presentation (or 1 sec after stimulus presentation) in the contralateral and bilateral stimulation conditions. The units extracted through each selection step were pooled together, creating a population of bilaterally responsive units and separating it from the remaining contralaterally responsive units (Figure 2). All further analyses were run separately on the two populations.

#### Statistics

Non-parametric statistics were used for all analyses performed with R^78^ (RStudio with the packages: "rcompanion"; "FSA"; "ggplot2"; "tidyverse"). For each dependent variable, we analysed the effects of “treatment” (light incubation), “hemispheres”, and their interactions using the Scheirer-Ray-Hare test. To obtain the overall effect size (eta-squared estimate η^2^), we calculated it based on the Kruskal-Wallis test for single significant factors, since the Scheirer-Ray-Hare test is an extension of the Kruskal–Wallis test. For this, we used the function “kruskal_effesize” from the “rstatix” package in R. The eta-squared estimate assumes values from 0 to 1, and, multiplied by 100 indicates the percentage of variance in the dependent variable explained by the independent variable. For post-hoc analysis of significant interactions between the two factors, we used Dunn's Kruskal-Wallis test for multiple comparisons (Dunn 1964). The dependent variables were: the spontaneous firing rate in the 1 sec before stimulus onset, averaged over all the stimulation conditions (contra-, ipsi- and bilateral); the firing rate peak occurring during the 1 second of stimulus presentation (ON peak); the latency of the ON peak (measured as the middle of the bin in which the peak occurred); the firing rate peak occurring during the 1 sec after the end of the stimulus presentation (OFF peak); the latency of the OFF peak. For the peak firing rates analysis, we subtracted the spontaneous firing rate. For figure representation, the asterisks indicate significant differences (∗<0.05, ∗∗<0.01, ∗∗∗<0.001).
